# Impact of age, sex, body constitution, and the COVID-19 pandemic on the physical fitness of 38,084 German primary school children

**DOI:** 10.1038/s41598-025-95461-5

**Published:** 2025-04-02

**Authors:** Florian Bähr, Toni Wöhrl, Paula Teich, Christian Puta, Reinhold Kliegl

**Affiliations:** 1 Faculty of Educational Sciences, Division of Sports and Movement Sciences, University of Erfurt, Nordhäuser Straße 63, 99089 Erfurt, Germany; 2 Faculty of Human Sciences, Department of Sports and Health Sciences, University of Potsdam, Potsdam, Germany; 3 Department of Sports Medicine and Health Promotion, Friedrich-Schiller-University of Jena, Jena, Germany; 4 Center for Interdisciplinary Prevention of Diseases Related to Professional Activities, Jena, Germany; 5 Center for Sepsis Control and Care (CSCC), Jena University Hospital/ Friedrich-Schiller- University Jena, Jena, Germany

**Keywords:** Public health, Physiology, Anatomy

## Abstract

Physical fitness (PF) is a vital health indicator, but many children do not meet the WHO physical activity guidelines. Low PF in children raises the risk of non-communicable diseases (NCDs) and negatively impacts their quality of life, a situation amplified during the COVID-19 pandemic. This study uses data from 38,084 German third-graders (7.8 to 9.4 years) across seven cohorts (school years 2017/18–2023/24) who participated in a statewide fitness evaluation program in the Federal State of Thuringia. We aimed to examine age, sex, and pandemic effects on endurance (6-minute run), coordination (star run), speed (20-meter linear sprint), lower limb power (powerLOW; standing long jump), upper limb power (powerUP; ball-push test), and static balance (one-legged stance with eyes closed). Data on height and mass was incorporated to clarify the task-specific impact of the body mass index (BMI) on performance. Our results corroborated reported age and sex effects, demonstrating linear development across the age range for all tests, and in static balance only, girls scored higher than boys. The relationship between BMI and task-specific performance revealed an inverted U-shaped function in weight-bearing tests (first four). Functions were steeper for boys than girls, implying a stronger BMI impact on performance for boys. BMI and age interaction suggest that being overweight may limit age-related performance benefits. Negative pandemic declines in endurance, coordination, powerLOW, and powerUP were more pronounced in “fitter” schools. In conclusion, task-specific performances should be interpreted considering age, sex, and body constitution. Pandemic-related performance declines emphasize the role of access to physical activity resources for all children.

**Supplementary Information:**

The online version contains supplementary material available at 10.1038/s41598-025-95461-5.

## Introduction

Physical fitness (PF) is an essential health indicator and a valid measure of activity-related bodily functions^1^. It encompasses health- and skill-related components such as cardiorespiratory endurance, muscle strength, speed, coordination, and balance^2^. Adequate cardiorespiratory and muscular fitness is associated with a lower risk of developing several non-communicable diseases (NCDs), which are the leading causes of death worldwide^3^. Being overweight and obese during childhood and adolescence significantly reduces quality of life^4^. Moreover, childhood obesity significantly elevates the risk of being overweight or obese for life^5–8^. However, maintaining a healthy fitness level during these critical developmental years is associated with a more favorable body composition and cardiometabolic profile in adulthood^9–12^.

Studies focused on PF trends of the last decades recently reported that about 80% of children and adolescents worldwide do not achieve the minimal daily physical activity goals the World Health Organization recommends^13–16^. In Germany, 72.5% do not meet them, and in the German region we examined (Federal State of Thuringia), that number is high at 69%^17^. Since the mid-1980s, a worldwide downward trend in cardiorespiratory fitness among children and adolescents has paralleled the increasing rates of overweight and obesity that started in the mid-1970s^18–20^. In Germany, both trends appear to have slowed down recently^18,20–23^. However, they leveled off at a higher number of overweight children and a lower level of cardiorespiratory fitness among children.

Recently, the COVID-19 pandemic has contributed to another increase in the number of overweight children in Europe^24–30^. In contrast, the impact on PF components was inconsistent and dependent on the analyzed sample size, the type of the observed living area (urban or rural), socioeconomic background, and pre-pandemic secular trends^24,25,27,29–38^. A representative German large-scale study^36^ on third-graders quantified the pandemic’s impact on physical fitness components in developmental losses of approximately five months for cardiorespiratory endurance, three months for coordination, one month for speed, and two months for upper limb muscle power. Gains of one month in lower limb muscle power and seven months in static balance were noted. Still, pre-existing secular trends influenced some of these changes, particularly regarding muscular power and balance.

It is well known that performance in fitness tests is strongly related to body constitution. In particular, the body mass index (BMI; in kg/m^2^) has proven to be a consistent and practical tool to classify the mass-to-height ratio and describes the relationship to PF components in an inverted U-shaped curve^29,39–46^. The precise shape of the function depends on the fitness component and varies depending on age and sex. For example, boys tend to exhibit sharper parabolic curves when BMI is associated with composite fitness scores, cardiorespiratory endurance, lower limb muscle power, or flexibility^39,41,42^. Thus, besides age and sex, body constitution is also an indispensable factor in assessing children’s performance for PF components. However, there is an ongoing debate regarding the reliability of BMI, waist-to-height ratio (WHtR), and waist circumference as predictors of childhood obesity, which currently favors WHtR^47–50^. Although the WHtR is a more reliable predictor, applying the measuring tape can trigger feelings of shame and fear of being touched among pupils in a non-clinical context. In contrast, height and weight are commonly known, and parental self-reports are recommended to classify the current BMI^51–53^, which encouraged its use in the present study.

Recent studies from a German region (i.e., the Federal State of Brandenburg) examined age and sex effects on six PF components in third-graders^36,54^. In addition, one of the studies demonstrated the impact of the COVID-19 pandemic on these components while adjusting for secular trends^36^. The present study aimed to replicate these previously examined age and sex effects on third-graders in another region of Germany (i.e., the Federal State of Thuringia) using the same test battery as the reference project^36,54^to assess PF components. Additionally, as parents reported the height and weight of their children in the present study, we went beyond the reference project. We incorporated the anthropometric covariates zBMI, zHeight, and zMass (i.e., anthropometric measures adjusted for age and sex using the LMS method^55–57^) to test their role in moderating sex, age, and COVID-19-related effects.

Specifically, we tested 38,084 third-grade primary school children from seven cohorts in the school years 2017/18–2023/24, which includes the start and end of the COVID-19 pandemic. The assessment of cohort 2019 extended into the year 2020 but was finished before pandemic-related school closures and other restrictions of movements took hold. As the assessment of cohort 2020 started only in the fall, all children of this cohort had experienced pandemic-related restrictions. We specified a regression discontinuity design (RDD) in a linear mixed model (LMM) to test whether there was a significant change in performance at the “critical date” 2020-08-30 (i.e., the first day of school in third grade for the 2020 cohort) and whether pre-pandemic secular trends differed from those after the critical date.

## Methods

### Sample and study design

This cross-sectional study was part of the “Bewegte Kinder = Gesündere Kinder” (BeKiGeKi) program, which was mandated and approved by the Ministry of Education, Science, and Culture of the Federal State of Thuringia, Germany. Participation in the statewide fitness evaluation program was obligatory for children in the third grade. The program assesses performance using the same tests as the Brandenburg EMOTIKON project^36,54^. The Ministry informed parents about the tests in advance, and their written consent was obtained. The research was conducted in accordance with the latest Declaration of Helsinki^58^.

During the school years 2017/18 to 2023/24, the schools reported data on 50,440 children. A “cohort” refers to the data from one school year, resulting in seven cohorts. Assessments were carried out for the 2017–2023 cohorts, covering the period from November 20, 2017, to December 21, 2023. All children of the pre-pandemic cohorts (2017–2019) were assessed before the first day of school closure on March 17, 2020. All children in pandemic cohorts (2020–2023) experienced school closure before their assessment.

We excluded children without information about age or sex (*n* = 438), who were older than nine years on August 1 (*n* = 8,660; i.e., those who had been enrolled a year later or had repeated a class), and who had turned eight years on or after January 1 of the following year (*n*= 80; i.e., very young children). In other words, we retained children who started school according to the legal key age^59,60^: those whose eighth birthday was on or before August 1 and younger children who turned eight between August 1 and December 31. Next, 50 children were excluded because their test scores were larger or smaller than 3 SD from the mean (exception: one-legged stance test; see below). Finally, parents of 3,178 children did not provide information about their children’s body height and mass. Data from the remaining 38,084 children (18,768 boys) were included in the analysis. The children’s mean age of the final sample at the test date was 8.9 years (*SD* = 0.3), ranging from 7.8 to 9.4 years.

### Physical fitness tests, covariates, and transformations

Children were assessed using the EMOTIKON test battery^54^. The six tests measure endurance (6-minute run), coordination (star run), speed (20-meter linear sprint), lower limb power (powerLOW; standing long jump), upper limb power (powerUP; ball-push test), and static balance (one-legged stance with eyes closed). Qualified physical education teachers at each school received a standardized test manual. They administered the tests according to the standardized testing protocols during regular physical education classes in the participating schools (see www.uni-potsdam.de/en/emotikon/projekt/methodik for further information on the test protocols). Before testing, all third graders performed a warm-up program consisting of running exercises (e.g., side steps) and small games (e.g., playing tag) for about 12 min. Several participants completed the tests station-by-station, each group in a different order. The 6-minute run was performed at the end with all participants. The tests were not allowed to be practiced in advance.

#### Endurance

Endurance was assessed using the 6-minute run. Children had to run as far as they could within six minutes at a self-paced velocity around an official volleyball field (9 × 18 m, with a pylon every 9 m; the marker was set beside the running court, resulting in six pylons around the field). The analysis utilized the maximal distance achieved during the six minutes in meters, rounded to the nearest nine-meter marker, as the dependent variable. If children stopped between two pylons at the stop signal, they were allowed to continue to the next pylon. The 6-minute run demonstrated reliability (test-retest) in children aged 7–11 years, with *r*= .92^61^. The 6-minute run correlated at *r* = .69 (*p* < .01) with VO2_max_assessed via gas analysis during a progressive treadmill test in children aged 9–11 years^62^.

#### Coordination

Coordination under time pressure was tested using the star run. Children had to complete a course with various movement directions and forms (i.e., running forward, running backward, side steps to the left, side steps to the right). The course had to be completed in a specified order over a 9 × 9 m star-shaped area where a pylon marks each of the four spikes. After starting in the center of the star, children had to complete the course as quickly as possible by running in every movement form twice within the given sequence. They had to touch each pylon with their hand. The total distance of the parkour was 50.912 m. Time was measured in seconds to the nearest 1/10 s. The fastest of the two test trials was used in the analysis. The star run demonstrated reliability (test-retest) in children aged 8–10 years, with an intra-class correlation coefficient (ICC) of 0.68^63^.

#### Speed

The 20-meter sprint assessed linear sprint speed. Children started the sprint from a standing position following an acoustic signal. Time was measured in seconds with 1/10 s accuracy. Each child had two trials, and the fastest trial was used for the analysis. The 20-meter sprint exhibited a test-retest reliability of *r*= .90 in children aged 7 to 11 years^61^.

#### PowerLOW

The standing long jump assessed lower limb muscle power (powerLOW). From a standing upright position, children were required to jump as far as possible with their feet parallel and in a shoulder-width stance. They were asked to jump with both legs and land with both feet. Children were allowed to swing their arms before and during the jump but were not allowed to touch the floor with their hands after landing. The distance between the children’s toes at takeoff and their heels at landing was measured to the nearest 1 cm. Children completed two test trials, and the trial with the better jump distance was used for analysis. The standing long jump demonstrated a test-retest reliability (ICC) of *r*= .94 in children aged 6–12 years^64^.

#### PowerUP

The ball-push test assessed upper limb muscle power (powerUP). Children were asked to hold a 1 kg medicine ball in front of their chests with their arms bent and then to push it as far as possible using both hands. The distance was measured in meters to the nearest 10 centimeters. The children completed the ball-push test twice, and the trial with the longest distance was used for analysis. In children aged 8–10 years, the ball-push test demonstrated a test-retest reliability (ICC) of *r*= .81^65^.

#### Static balance

The one-legged stance with eyes closed assessed static balance. The children’s standing leg was slightly bent, with both knees pointing forward. The free leg was bent between 60° and 90° at the hip joint and approximately 90° at the knee joint. The children’s hands were held akimbo. They were asked to close their eyes and remain in this position for as long as possible. Time was measured with 1 s accuracy. The maximum duration of a trial was set at 60 s. If a child’s test trial lasted less than 5 s, they were granted another trial. For the analyses, scores greater than 60 s, indicating that the test had not ended in time, were set to the maximum value of 60 s. The one-legged stance test with eyes closed demonstrated test-retest reliability (ICC) of *r*= .69 in children aged 7–10 years^66^.

#### Transformation of physical fitness measures

As was the case for the data in the EMOTIKON project, Box-Cox distributional analysis^67^ suggested reciprocal transformations of star run time and 20-meter sprint time to speed scores (i.e., dividing the run distance by time) and a logarithmic transformation of one-legged stance time. These transformations showed that higher scores represent better performance across all scores. To identify outliers, z-scores were computed for each test, separately for boys and girls. For each test, except for the one-legged stance test, *z*-scores outside of a ± 3 SD range were identified as outliers and removed. Since the one-legged stance test was terminated after a maximum of 60 s, test scores exceeding 60 s were deemed impossible. The entire range of scores for the one-legged stance test indicated valid performance; therefore, we did not apply the ± 3 SD criterion to this test. Finally, z-scores were recomputed for each test, this time aggregated over boys and girls to account for sex-related differences in the data.

#### Covariates of BMI, body height, and body mass

The children’s parents voluntarily self-reported body height and mass in centimeters and kilograms. This approach was required to protect children’s data privacy. All three covariates correlate strongly with age and sex. Therefore, sex- and age-adjusted *z*-scores for mass, height, and BMI (in kg/m^2^) were retrieved from the R package *childsds*^68^. These z-scores (also known as BMI-SDS, Mass-SDS, and Height-SDS) were computed with reference to anthropometric tables of a large representative sample of German children^69^using the LMS method^55–57^. The LMS estimates three parameters from smoothed percentile growth curves: the median (M), the generalized coefficient of variation (S), and the Box-Cox power transformation (L). The BMI z-score is calculated using the following formula:

1 $$\text{zBMI} = ((\text{BMI}/\text{M})^{L}-1) / (\text{L} \times \text{S})$$

This allows for converting BMI values into standardized scores that reflect how far a child’s BMI deviates from the median for their age and sex. Analogously, z-scores are computed for mass and height. The children of the present study were average weight (zMass = 0.01) but taller (zHeight = 0.20). Consequently, they had a smaller zBMI (−0.14) than the representative German sample.

Powers of z-scores are no longer z-scores. However, their quadratic and cubic terms can be transformed into z-scores with M = 0 and SD = 1 using the following three formulae:

2 $$\text{zBMI2} = (\text{zBMI}^{2} - 1) / \text{sqrt(2)}; \text{because}\, \text{mean}:\text{E}[\text{x}^{2}]=1\, \text{and}\, \text{variance}: \text{Var}(\text{x}^{2}) = 2.$$

3 $$\text{zBMI3} = \text{zBMI}^{3} / \text{sqrt(15)}; \text{because}\, \text{mean}: \text{E}[\text{x}^{3}]=0\, \text{and}\, \text{variance}: \text{Var}(\text{x}^{3}) = 15.$$

4 $$\text{zHeight2} = (\text{zHeight}^{2} - 1) / \text{sqrt}(2)$$

Second- and third-order polynomial terms were transformed accordingly before being included as covariates in the LMM.

### Statistical analysis

#### Regression discontinuity design (RDD)

Data were collected from three pre-pandemic cohorts, assessed between November 20, 2017, and March 6, 2020, and four pandemic cohorts, all of whom experienced school closures (albeit in different grades) between their first day at school on August 30, 2020, and December 21, 2023. There are two potential sources of the difference between pre-pandemic and pandemic cohorts. Changes in test performance could be a consequence of the COVID-19 pandemic but could also result from pandemic-independent secular trends^54^. For example, a simple linear decline in performance from 2016 to 2023 yields a significant but possibly spurious effect attributed to the COVID-19 pandemic. Thus, evidence for a negative pandemic effect requires (1) a negative step at the critical date or (2) a negative change in the slope of performance during the pandemic years compared to the pre-pandemic years starting at a critical date.

The RDD^70,71^ provides test statistics for a pandemic effect at a critical date and for secular trends before and after this date. In our implementation, we fixed the critical date as the first day of school in third grade for the 2020 cohort (i.e., 2020-08-30), dummy-coded pre-pandemic (0) and pandemic (1) cohorts, and centered dates of assessment at the critical date. This specification allows us to estimate the parameters of the pre-pandemic and pandemic regression lines. Intercepts for both regression lines are estimated at the critical date, with the difference between these intercepts estimating the size of the pandemic effect at the critical date. A significant difference between intercepts provides evidence of discontinuity at the critical date. Conversely, in the previously mentioned example of a straight linear decline across all years, pre-pandemic and pandemic slopes will be identical at the critical date. Therefore, according to the RDD analysis, there will be no evidence of a pandemic effect at the critical date despite the difference between the means for pre-pandemic and pandemic assessments.

There is also a second, usually weaker test for a pandemic effect. The regression coefficient for the interaction of the dummy-coded pandemic variable and assessment date (centered at the critical date) tests a difference between pre-pandemic and pandemic regression slopes. In the above example, a significant negative interaction coefficient represents a stronger decline in performance for the pandemic than in pre-pandemic assessments. In our parameterization of the RDD, we directly estimated both slopes. Unfortunately, given the well-known limitations associated with quasi-experimental designs and depending on the specifics of the profile, an RDD may still overestimate or underestimate the true effect size associated with the critical date. There is usually much room for ambiguity in interpretation, especially in the absence of clear evidence for a discontinuity (step) at the critical date.

#### Linear mixed model (LMM)

We implemented the RDD in an LMM. Along with variables coding for the test date and cohort/COVID-19 pandemic-related effects and slopes, covariates of age (centered at 8.9), sex (sequential difference contrast with positive estimates indicating better performance of boys), zMass, zHeight, zHeight2, zBMI, zBMI2, and zBMI3, and interactions among them were considered fixed effects. Fixed effects were specified as nested under the six tests: endurance, coordination, speed, powerUP, powerLOW, and static balance.

Child and school were included as random factors. The random-effect structure (RES) included variance components (VCs) and correlation parameters (CPs) for the six tests for both random factors. Additionally, school-related VCs and CPs for the effects of age, sex, cubic zBMI, and the three RDD-based effects were also considered for this random factor.

The selection of a parsimonious LMM followed the recommendations of Bates et al.^72^. Parsimonious model selection maintains the statistical power^73^and determines CPs that may be needed to interpret fixed effects^74^. The data did not support the LMM with the most complex RES (i.e., the model was singular for the RES parameter vector). VCs for cubic zBMI were estimated to be zero or exhibited very small values (relative to the residual VC); they and their associated CPs could be removed without a significant decrease in the goodness of model fit according to the AIC/BIC statistics. This reduction in model complexity resulted in an RES supported by the data (see Table 3)  in the Results section. Fixed effects were never inspected during this series of iterations. Therefore, their significance or non-significance did not influence the selection of RES.

The same fixed-effect structure was kept for all six tests; there was no selective or component-specific removal of non-significant terms. The fixed-effect part of the LMM always included effects of sex, age, zBMI, zBMI2, zBMI3, the COVID-19 pandemic effect, and two linear trends for pre-pandemic and pandemic cohorts. Additionally, we considered incorporating fixed effects of age^2, zMass, zMass2, zHeight, zHeight2, and multiplicative interactions involving at most two or three of these thirteen terms. Using a drop of five AIC units as a criterion, zMass, zHeight, zHeight2, sex x zBMI, sex x zBMI2, age x zBMI, sex x COVID-19 effect, and age x COVID-19 effect were also kept in the LMM (see Supplementary Table 1).

The final LMM estimated 163 model parameters (i.e., 96 fixed effects, 15 VCs, 51 CPs, and 1 residual VC) for 221,252 performance measures from 38,084 children in 465 schools. There are 118,177 effective (geometric) model degrees of freedom and 103,075 (= 221,252 − 118,177) effective residual degrees of freedom. The geometric degrees of freedom represent the sum of the leverage values; they correspond to the rank of the model matrix, also known as the trace of the hat matrix.

The usual diagnostic plots of model residuals did not reveal any serious violations of model assumptions or outliers (see Supplementary Fig. 1). To clarify, we estimated the final LMM with an alternative parameterization to test the significance of the change between pre-pandemic and pandemic slopes.

LMMs were estimated and post-processed with MixedModels.jl (v4.26.1)^75^, MixedModelsExtras.jl (v2.1.1)^76^, and MixedModelsMakie (v0.4.4)^77^in the Julia programming language (v1.11.1)^78^in the VS Code IDE (v1.95.0; Microsoft Corporation, WA). For data analyses and graphics, we used mainly tidyverse (v2.0.0)^79^and easystats (v0.7.3)^80^packages in the R language (v4.4.1)^81^along with the RStudio IDE (v2024.09)^82^. Data, Julia, and R scripts are available in the OSF repository https://osf.io/ztyfp/. They allow for the reproduction of figures and analyses. Specifically, LMM model objects can be quickly restored from parameter files located in the OSF repository’s *fits* folder.

## Results

### Descriptive statistics

Table 1 reports the demographics and descriptive statistics for body constitution measures for the final sample of 38,084 children (18,768 boys; 19,316 girls). The data indicate a notable increase in the number of participating schools and, by implication, the number of children tested each year, with a decrease in 2020 (the first year of the COVID-19 pandemic).

**Table 1:** Demographics and descriptive statistics for body constitution by sex, pre-pandemic, and COVID-19 pandemic cohorts.

		Cohorts 2017–2019		Cohorts 2020–2023	
		N		N	
Schools (distinct per year)		243 (49; 174; 193)		459 (77; 190; 396; 424)	
Children	Boys	5,340 (= 644 + 2,122 + 2,574)		13,428 (= 905 + 2,142 + 4,641 + 5,740)	
	Girls	5,498 (= 704 + 2,176 + 2,618)		13,818 (= 952 + 2,259 + 4,815 + 5,792)	

		M	SD	M	SD
Age [years]	Boys	8.9	0.3	8.8	0.3
	Girls	8.9	0.3	8.8	0.3
Height [cm]	Boys	137	6.6	137	6.6
	Girls	136	6.5	135	6.6
Mass [kg]	Boys	31.7	6.7	32.0	6.9
	Girls	31.2	6.7	31.0	6.7
BMI [kg/m^2^]	Boys	16.9	2.7	17.0	2.8
	Girls	16.9	2.8	16.8	2.8

Means and standard deviations of body constitution (i.e., height, mass, and the derivative measure BMI (in kg/m²)) did not vary significantly across the cohorts.

Table 2 provides statistics for the six tests in their original metric, broken down by sex for pre-pandemic and pandemic cohorts. The t-statistics in the rightmost column (ΔCohorts) indicate the effects of the COVID-19 pandemic by sex. Their magnitudes reflect relative effect sizes and are noticeably larger for the three running tests (6-minute run, star run, 20-meter sprint) than for the other three tests; for powerUP (ball-push test), the difference is positive for boys but negative for girls. Static balance (one-legged stance test) was positive for both boys and girls (i.e., performance was better for the pandemic than the pre-pandemic cohorts).

**Table 2:** Physical fitness statistics by component, sex, pre-pandemic, and pandemic cohorts.

Component Test		Cohorts2017–2019			Cohorts 2020–2023			ΔCohorts		
		N	M	SD	N	M	SD	Δ	t	p
Endurance [m]	Boys	5,199	1016	142	13,009	983	145	−33	−14.3	< 0.001
(6-minute run)	Girls	5,375	946	122	13,395	921	125	−25	−12.3	< 0.001
Coordination [m/s]	Boys	4,896	2.01	0.31	12,355	1.97	0.31	−0.04	−7.9	< 0.001
(Star run)	Girls	5,083	1.94	0.28	12,921	1.89	0.28	−0.05	−10.6	< 0.001
Speed [m/s]	Boys	5,249	4.64	0.41	13,221	4.57	0.41	−0.07	−10.3	< 0.001
(20-meter sprint)	Girls	5,423	4.52	0.39	13,583	4.45	0.38	−0.07	−11.1	< 0.001
PowerLOW [cm]	Boys	5,265	132	20	13,203	131	20	−1.39	−4.2	< 0.001
(Standing long jump)	Girls	5,429	125	19	13,613	123	19	−1.83	−6.0	< 0.001
PowerUP [m]	Boys	5,285	4.06	0.70	13,192	4.10	0.71	0.04	3.3	0.0016
(Ball-push test)	Girls	5,436	3.62	0.62	13,595	3.60	0.63	−0.02	−2.3	0.0239
Balance [log(sec)]	Boys	5,038	2.44	0.75	12,820	2.49	0.84	0.05	4.1	< 0.001
(One-legged stance)	Girls	5,284	2.61	0.78	13,383	2.70	0.84	0.09	7.3	< 0.001

The t-statistics in the rightmost column show the differences between the pre-pandemic (2017–2019) and pandemic cohorts (2020–2023).

### Inferential statistics

**Fig. 1:**
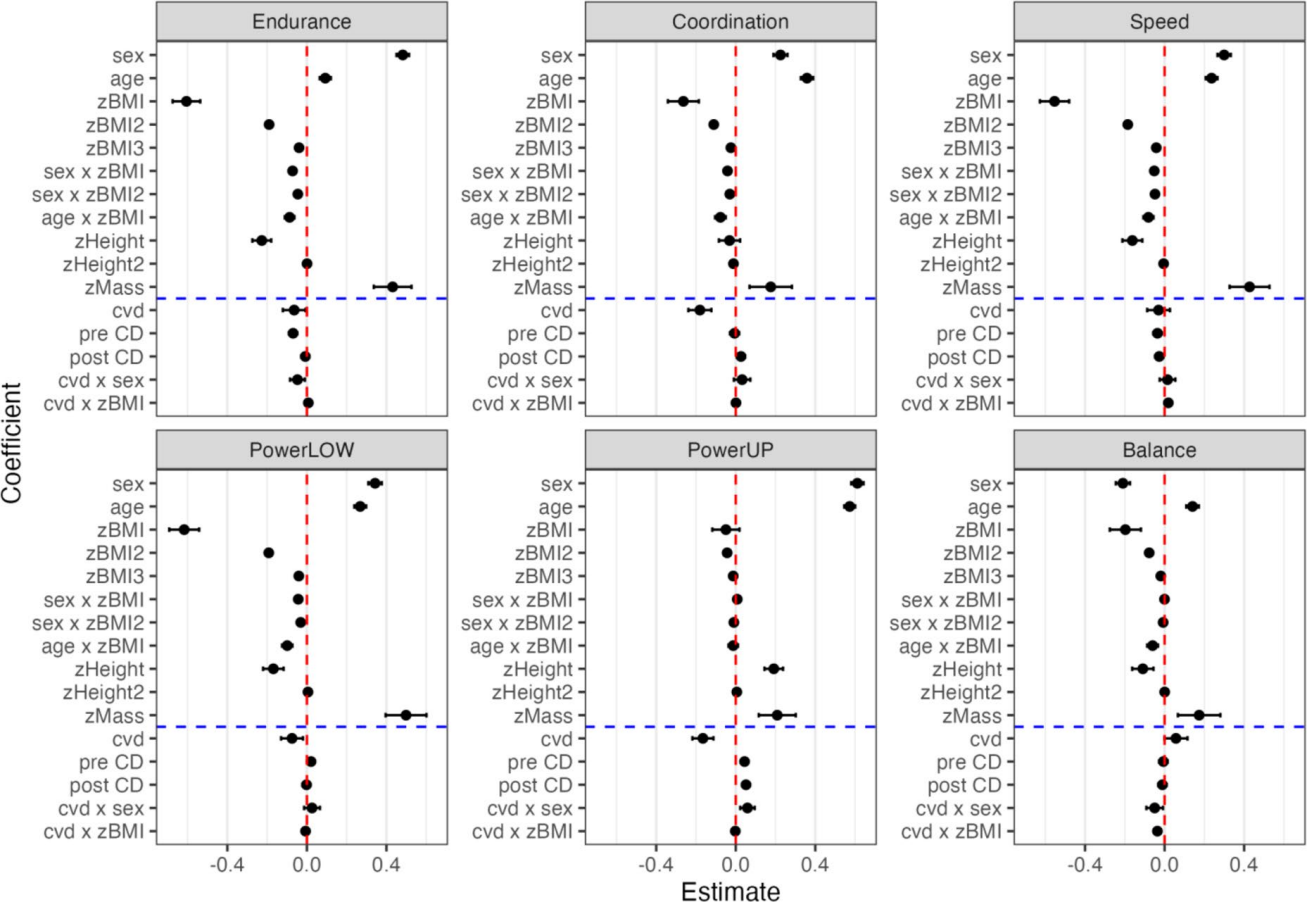
LMM fixed effects in six physical fitness components. Endurance = 6-minute run, Coordination = star run, Speed = 20-meter sprint, PowerLOW = standing long jump, PowerUP = ball-push test, Balance = one-legged stance. LMM is based on random factors: school (*n* = 465) and child (*n* = 38,084); the number of observations: 221,252. Error bars (not always visible) represent 95% CIs.

In the pre- and post-comparisons of Table 2, the effects of the COVID-19 pandemic were confounded by secular trends and possibly masked by differences in sex, age, body constitution, schools, and children. In the RDD-based LMM, linear cohort trends before and after the pandemic’s onset were considered when estimating the effects of the COVID-19 pandemic, along with effects from other potential covariates. RDD specifications and model selection are described in the Methods section and documented in the OSF repository (https://osf.io/ztyfp/). Dependent variables for all analyses are test-specific z-scores. All fixed-effect coefficients and their 95% confidence intervals (CIs) are displayed in Fig. 1. The coefficients and their associated test statistics that this figure is based on are presented in Supplementary Table 1. The effect sizes of specific effects can be compared across components since all test scores are z-scores (i.e., M = 0 and SD = 1). However, as effects were specified as nested within physical fitness components in the LMM, there is no explicit test for the significance of differences between tests. The effect sizes of anthropometric covariates can be compared within components because zBMI, zBMI2, zBMI3, zMass, zHeight, and zHeight2 are z-scores.

### Sex and age differences

**Fig. 2:**
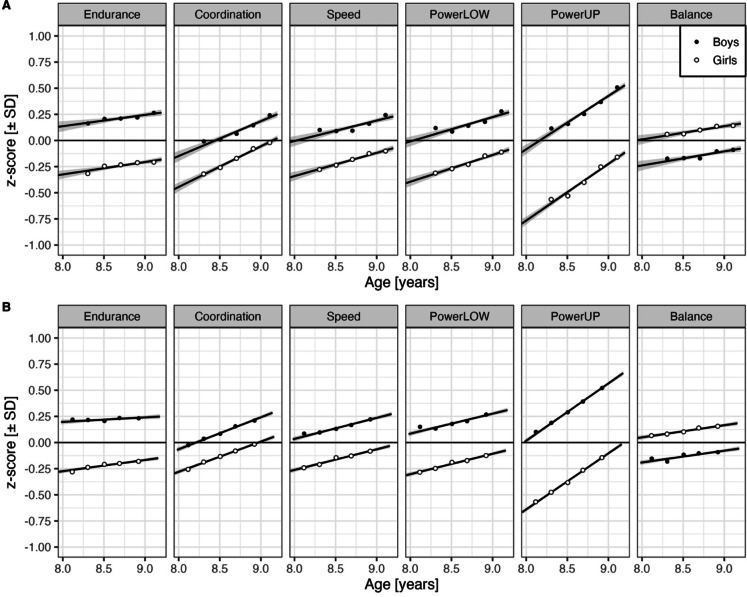
Age and sex effects on six physical fitness components for third-grade elementary school children from (A) the 2017–2023 cohorts in Thuringia (*N* = 38,084 children) and (B) the 2016–2022 cohorts in Brandenburg (*N* = 98,510 children). Endurance = 6-minute run, Coordination = star run, Speed = 20-meter sprint, PowerLOW = standing long jump, PowerUP = ball-push test, Balance = one-legged stance. Points represent means for 0.20-year-wide bins; smooths are linear fits to observations. Error bands for smooths indicate 95% CIs.

Sex and age effects were highly similar to those from the Brandenburg project^36,54^ (see Fig. 2, panel A for Thuringia and panel B for Brandenburg). Boys scored significantly higher than girls in endurance, coordination, speed, powerLOW, and powerUP but significantly lower on static balance (Fig. 2). The age effects demonstrate a significant positive linear development across the age range for all tests. Notably, the relative magnitude of age effects (slopes) and sex differences were the same in the present study compared to the Brandenburg project^36,54^. Remarkably, there was no significant quadratic age effect and no significant age x sex interaction despite an abundance of statistical power (i.e., ΔAIC for models ≤ 5 with these terms included) in these two studies. Therefore, these terms were removed from the final LMM and are not shown in Fig. 1. Supplementary Fig. 2 (panel A) shows that partial fixed effects of age and sex agree very well with zero-order relations in Fig. 2A.

### Body mass index

**Fig. 3:**
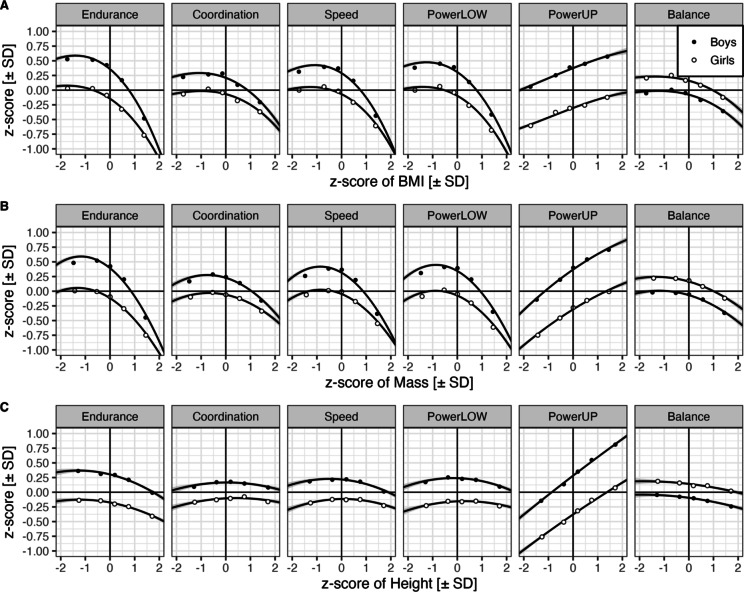
Zero-order fixed effects for the interaction of sex and (A) cubic trend effects of zBMI, (B) quadratic z-height, and (C) quadratic z-mass fit to observations. Endurance = 6-minute run, Coordination = star run, Speed = 20-meter sprint, PowerLOW = standing long jump, PowerUP = ball-push test, Balance = one-legged stance. Points represent observed z-score means for bins with approximately equal numbers of children. The interaction with sex is significant for the linear and quadratic trends of zBMI.

For the first four weight-bearing tests (i.e., activities that the skeletal system does against gravity, i.e., endurance, coordination, speed, and powerLOW), the relationship with zBMI was characterized by an inverted U-shaped cubic function (zBMI + zBMI2 + zBMI3; see Fig. 1 for effect sizes of the three coefficients and Fig. 3A for the zBMI zero-order relations). Specifically, as expected, scores were highest for children in the low to middle zBMI range, decreased slightly with decreasing zBMI, and declined significantly with increasing zBMI. The curve peaks are consistently below the reference zero line. Figure 3A also reveals that for these four tests, zBMI curves are not parallel for boys and girls, but sex differences converge for high zBMI, implying a stronger BMI impact for boys than for girls. Sex interactions with zBMI and zBMI2 terms were significant (see 95%-CIs in Fig. 1 and test statistics in Supplementary Table 1). The primary source of zero-order relations are zBMI-, not correlated zMass- or zHeight-coefficients. Except for endurance, zBMI tends to overestimate performance for low and underestimate performance for high values (see Supplementary Fig. 3A for corresponding partial fixed effects).

PowerUP and static balance exhibited distinctly different zBMI profiles. For powerUP, performance increased with increasing zBMI and did not significantly interact with age or sex; that is, the curves were statistically parallel for boys and girls, as well as for younger and older children in the third grade. The zBMI curve was also inverted U-shaped for static balance, but girls outperformed boys. As for powerUP, there was no significant interaction between zBMI and sex.

**Fig. 4:**
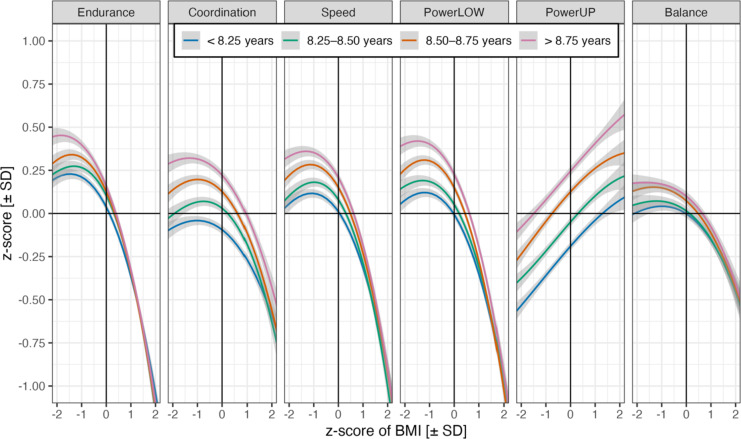
Zero-order fixed effects for the interaction of age and cubic-trend effects of zBMI on observations from four age groups). Children were aggregated into four age groups to visualize the interaction; in the linear mixed model (LMM), age was modeled as a continuous linear covariate and interacted significantly with the linear trend of zBMI. Endurance = 6-minute run, Coordination = star run, Speed = 20-meter sprint, PowerLOW = standing long jump, PowerUP = ball push test, Balance = one-legged-stance.

Figure 1 and Supplementary Table 1 also present significant interactions between age and linear zBMI for all tests except powerUP. In these tests, positive age differences are visible for low zBMI values, which decreased and vanished as zBMI increased (see zero-order smooths in Fig. 4; values were aggregated into four age groups for visualizing the interaction; corresponding partial fixed effects are shown in Supplementary Fig. 4). There is no evidence of such convergence for powerUP.

### Body mass and height

BMI is computed from body mass and height (in kg/m^2^). Thus, correlations exist between zBMI and zMass (*r* = .86), between zBMI and zHeight (*r* = .23), and between zMass and zHeight (*r* = .54). Consequently, zBMI curves are very similar to zMass curves (see Fig. 3B). However, in the LMM, the impact of zMass was notably weaker than that of cubic zBMI; specifically, only the linear effect of zMass showed a strong positive correlation across all six tests (see corresponding plots of partial fixed effects in Supplementary Fig. 3). Removing zMass from the LMM caused a significant loss in the goodness of fit [Δχ^2^(6) = 147.2, *p* < .0001]. There was no reliable evidence for a quadratic trend of zMass [Δχ^2^(6) = 7.2, *p* = .3051]. As such, this term was not included in the LMM.

The quadratic zHeight also exhibited inverted but much shallower U-shaped curves for five of the six tests. Overall, zHeight2 was significant [Δχ^2^(6) = 15.1, *p* = .0196] and was included in the LMM. None of the sex- and COVID-19-related terms interacted significantly with zMass and zHeight, meaning that the AIC decreased by less than five units or even increased when these terms were added to the LMM for all model comparisons. Thus, zMass and zHeight act as “correction” factors (i.e., suppressor variables) for the dominant cubic zBMI (see corresponding plots of partial fixed effects in Supplementary Fig. 3). Qualitatively different from the other five tasks, the effect of zMass and zHeight2 aligned with the zero-order increase in the performance of powerUP across their entire ranges.

### Secular trends and COVID-19 pandemic effects

The RDD specification tested the effects of the COVID-19 pandemic on the first day of school in the 2020/21 school year (i.e., at the critical date, 2020-08-30). The RDD yields three fixed effects for each of the six fitness components (i.e., the difference between the two intercepts at the critical date, the linear pre-pandemic and pandemic slopes), adjusted for all other covariates, variance components (VCs), and correlation parameters (CPs; see below for details) in the LMM.

**Fig. 5:**
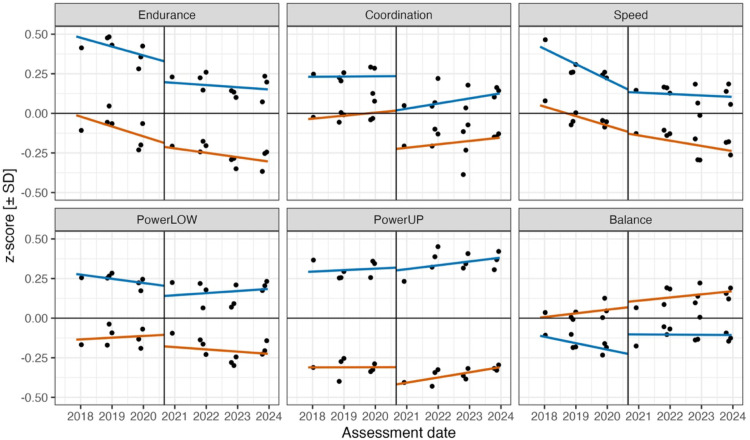
Zero-order relations for the regression of physical fitness on the assessment date (2017-11-20 to 2023-12-21) and the critical date for the test of the COVID-19 pandemic effect on 2020-08-30 (i.e. , the vertical line marks the first day of school in third grade for the 2020 cohort of boys , blue line , and girls , red line). Endurance = 6-minute run; Coordination = star run; Speed = 20-meter sprint; PowerLOW = standing long jump; PowerUP = ball-push test; Balance = one-legged stance. Black dots represent observed z-score means for bins of assessment dates with at least 1 , 000 children; green dots indicate mean model predictions for these bins.

Figure 5 displays the zero-order effects with separate linear trends for pre-pandemic and pandemic assessments by sex; corresponding plots of partial fixed effects are shown in Supplementary Fig. 5. The differences between the two lines at the critical date (i.e., the vertical line) represent the test-specific estimates of the COVID-19 pandemic effect. Points depict the means of bins containing at least 1,000 children and illustrate the variability across time

Significant negative effects of the COVID-19 pandemic were observed for endurance, coordination, powerLOW, and powerUP (all *p* < .05). For endurance and powerUP, the effects were moderated by interactions with sex. As shown in Fig. 5, for endurance, the effect was attributed to performance changes in boys (b = −0.05, z = −2.50, *p* < .05), while for powerUP, the effect was attributed to performance changes in girls (b = + 0.06, z = 3.16, *p* < .01). Additionally, the pandemic slopes (i.e., slopes between 2020 and 2023) were significantly more positive than the pre-pandemic slopes for endurance (Δb = + 0.06, z = 4.32, *p* < .01) and coordination (Δb = + 0.03, z = 2.04, *p* < .05). Evidence of pandemic effects and cohort trends is not conclusive. For instance, the endurance profile could also be consistent with a non-linear secular plateau of performance rather than a negative pandemic effect.

The pandemic effect was not significant for Speed, where a significant negative pre-pandemic slope (b = −0.04, z = −3.31, *p* < .01) was followed by a significant negative pandemic slope (b = −0.03, z = −2.57, *p* < .05). For static balance, there was a significant interaction between the pandemic effect and sex (b = −0.05, z = −2.32, *p* < .05), with a larger positive partial pandemic effect observed for girls, although the zero-order relations suggested the reverse.

### Child- and School-related variance components and correlation parameters

The LMM also estimated child-related and school-related VCs and CPs, as reported in Table 3. Component CPs represent correlations between test scores after adjustment for fixed effects, and VCs and CPs are estimated for the other random factors.

**Table 3:** Variance components and correlation parameters.

	VC	CP							
	SD	E	C	S	PL	PU	B	Covid @ CD	Slope_pre_
*Child*									
Endurance (E)	0.63								
Coordination (C)	0.73	+ 0.53							
Speed (S)	0.71	+ 0.60	+ 0.62						
PowerLOW (PL)	0.72	+ 0.54	+ 0.61	+ 0.77					
PowerUP (PU)	0.63	+ 0.42	+ 0.51	+ 0.59	+ 0.65				
Balance (B)	0.76	+ 0.18	+ 0.23	+ 0.22	+ 0.24	+ 0.20			
*School*									
Endurance	0.46								
Coordination	0.40	+ 0.35							
Speed	0.40	+ 0.43	+ 0.38						
PowerLOW	0.36	+ 0.51	+ 0.50	+ 0.58					
PowerUP	0.39	+ 0.46	+ 0.39	+ 0.51	+ 0.63				
Balance	0.40	+ 0.19	+ 0.23	+ 0.26	+ 0.37	+ 0.47			
Covid @CD	0.36	−0.12	−0.17	−0.14	−0.27	−0.34	−0.22		
Slope_pre_ CD	0.13	+ 0.57	+ 0.28	+ 0.48	+ 0.72	+ 0.71	+ 0.56	−0.28	
Slope_post_ CD	0.15	−0.26	−0.07	−0.20	−0.17	−0.09	−0.05	−0.78	−0.21

LMM estimates of variance components (VCs) and correlation parameters (CP) for the random factor Child. LMM is based on the random factors school (*n* = 465) and child (*n* = 38, 084); the number of observations is 221, 252. Residual SD = 0.51. Endurance (E) = 6-minute run; Coordination (C) = star run ; Speed (S) = 20-meter sprint; PowerLOW (PL) = standing long jump; PowerUP (PU) = ball-push test; Balance (B) = one-legged stance. Covid @ CD = Covid-19 pandemic effect at critical date. Slope_*pre*_ *CD/Slope*_*post*_ *CD = Cohort slope before/after the critical date.*

CPs between endurance, coordination, speed, powerLOW, and powerUP tests indicated moderate to high correlations when computed for children (range: 0.42–0.77) and for schools (range: 0.35–0.63)., Except for powerLOW, powerUP showed lower correlations with the other four fitness tests (ranges: 0.42–0.59 and 0.39–0.51 for Child and School, respectively). Thus, the correlational pattern reflected the qualitative differences in the tests’ relationships with the body constitution described above.

There were also reliable differences between schools regarding the size of the COVID-19 pandemic’s effect on the critical date and the linear trends before and during the pandemic. The negative correlations between performance and the pandemic’s effect indicate that the pandemic had a stronger impact on “fitter” schools. The positive correlations between the pandemic’s effect and the pre-pandemic slope suggest that fitter schools experienced less negative (or more positive) pre-pandemic trends. This correlation was significantly attenuated for pandemic cohorts.

Finally, the three COVID-related effects were negatively correlated. Positive (less negative) pre-pandemic cohort trends were associated with larger negative COVID effects at the critical date (−0.28) and with negative (less positive) cohort trends during the pandemic (−0.21). Schools exhibiting more pronounced negative COVID effects at the critical date were more likely to demonstrate positive (less negative) cohort trends during the pandemic (−0.78).

## Discussion

The study evaluates the fitness components of 38,084 third-graders (7.8 to 9.4 years) across seven cohorts (school years 2017/18–2023/24), analyzing the impact of age, sex, body constitution, and the COVID-19 pandemic on endurance (6-minute run), coordination (star run), speed (20-meter linear sprint), lower limb power (powerLOW; standing long jump), upper limb power (powerUP; ball-push test), and static balance (one-legged stance with eyes closed). We aimed to (1) replicate previously reported age and sex effects^36,54^, (2) analyze the impact of body constitution, and (3) evaluate the effect of the COVID-19 pandemic, accounting for secular trends.

### Replication of age and sex effects

Although replicating results is a well-known challenge in social science^83^, we could replicate previously reported age and sex effects in six fitness tests^36,54^. Developmental rates and the magnitude of sex effects, along with the lack of an interaction between age and sex and the absence of a quadratic age effect (despite an abundance of statistical power), closely matched those reported in the Brandenburg EMOTIKON project for the same six tests. Sex effects vary substantially between the six different tests. They are largest in the ball-push test assessing powerUP for obvious muscle-related reasons^84^ and smallest in the star run, which assesses coordination under time pressure (see Fig. 1). For further discussion of factors associated with age and sex differences in these six fitness tests, we refer to our previous reports^36,54,85^.

### Effects of body constitution

The data supported cubic zBMI trends for all six tests. BMI signifies the age- and sex-adjusted BMI based on a large, representative sample from Germany^69^. Similarly, we used age- and sex-adjusted zMass and height values. The effects of body constitution were similar in all weight-bearing tests (i.e., endurance, coordination, speed, and powerLOW). An attenuated zBMI curve characterized static balance. PowerUP exhibited a distinctly different BMI function.

#### Weight-bearing tests (6-minute run, star run, 20-meter sprint, and standing long jump)

Weight-bearing tests are activities the skeletal system does against gravity. Zero-order zBMI functions for endurance, coordination, speed, and powerLOW were characterized by an inverted U-shape. Performance peaked below the zBMI middle range and decreased to both sides, with a steeper decline in the direction of over- than underweight. The function was steeper for boys than girls in all four tests. This may be related to boys’ greater percentage of muscle and lean mass and lower fat mass compared to girls^86–88^. In contrast to fat, muscle is an active tissue that generates force. Changes in BMI, primarily due to boys’ muscle mass in the middle BMI range, may account for the greater impact of BMI on boys than on girls.

BMI interacted with age across all four tests, indicating that the age differences in test performance were largest within the low and middle BMI ranges and decreased with increasing BMI. Being overweight or obese may reduce the anticipated benefits of aging, leading to performance improvements that occur more slowly than expected over time.

Previous studies reported that taller, leaner children and adolescents perform better in endurance runs^89^, the 20-meter sprint^89^, and the standing long jump^90–92^. Similar to our findings, some studies testing the form of the relationship between fitness and BMI reported inverted U-shaped relationships between fitness tests and BMI^39,41,43–45,93^. For instance, Chen et al.^39^tested children between 7 and 9 years old and reported that parabolic BMI functions with a more pronounced peak in boys in tests, including an endurance run, 50 m dash, and standing long jump. Huang and Malina^93^tested children and adolescents aged between 9 and 18 years. They reported that for a fitness index (computed from sit-ups, standing long jump, sit and reach, and an endurance run), BMI curves were curvilinear for children between 9 and 10 years and became more parabolic with increasing age, with sharper peaks for boys than girls. Time to complete an 800/1000m run/walk increased linearly with BMI in boys and girls between 9 and 10.5 years^41^. Similarly, standing long jump performance decreased linearly with BMI for boys between 9 and 10.5 years. However, it was characterized by a parabolic curve (i.e., performance decreased after an initial increase and peak) for girls of the same age^41^.

In addition to cubic BMI trends, there were significant positive linear mass-SDS effects on all four fitness tests and negative linear height-SDS effects on performance in the 6-minute endurance run, 20-meter sprint, and standing long jump test. These partial effects differed from the zero-order relations shown in Fig. 3, which indicated inverted U-curves for mass, similar to those for BMI, and strongly attenuated U-curves for height. The reason for the divergence between zero-order and partial effects is that zBMI, zHeight, and zMass positively correlate with each other, especially with a high correlation between zBMI and zMass (*r* = .86). zHeight and zMass suppressed the variance in zBMI, which is unrelated to performance and served as correction terms for the cubic zBMI function. The positive partial effect of zMass in the present study may also indicate an additional effect of muscle mass that is not yet represented in the zBMI and that increases running and jumping performances.

#### One-legged stance test with eyes closed

 The one-legged stance performance exhibited a similar zBMI curve to the weight-bearing tests. However, the peak was less pronounced, indicating a smaller impact of zBMI changes on test performance. In contrast to the weight-bearing test, the function was statistically parallel for boys and girls. Regarding the 6-minute run, 20-meter sprint, and standing long jump, the estimate of linear height-SDS was negative, while for all fitness tests, the estimate of linear mass-SDS was positive. Additionally, age interacted with zBMI, indicating that smaller BMI levels were associated with smaller age gains.

#### Ball-push test

The ball-push test exhibited a qualitatively different zBMI function than the other five fitness tests. Zero-order zBMI functions indicated that performance increased linearly with increasing zBMI, as well as with increasing zMass and zHeight. Likely due to collinearity, the model estimates for zBMI were not significant. However, they were significantly positive for linear zMass and zHeight. The positive effect of zMass aligns with other studies indicating that overweight and obese children performed better than normal-weight children in these tasks^40,94^. Higher body mass increases inertia and may stabilize the child while throwing the ball.

Independent of BMI, taller children demonstrate better ball-push test performance due to their higher ball drop height. Hypothetically, although not practical in a school setting, upper limb muscle power could be measured directly via video recording, using an accelerometer on the ball, or calculated using the throwing angle, drop height, and throwing distance. To obtain a more valid estimate of children’s upper limb muscle power while maintaining practicality, children could throw the ball while sitting against a wall^32,43^ to compensate for the effect of inertia and minimize the impact of body height (i.e., drop height of the ball) on performance.

### COVID-19 pandemic effects and secular trends

Another goal was to examine the effects of the COVID-19 pandemic on performance in these six fitness tests. The comparison of test means indicates that performance was lower after the onset of the pandemic (2020–2023) than before (2017–2019) in almost all tests. The exception was the ball-push test, where only girls exhibited lower performance. At the same time, boys showed improved performance during the pandemic, and the one-legged stance, in which performance increased after the pandemic onset (see Table 1). However, since pandemic effects are confounded with secular fitness trends^18,20^, we used a regression discontinuity design^70,71^ to adjust for these cohort trends, testing the pandemic’s impact on the first day of school in the 2020/21 school year. At this critical date, pandemic effects were negative in the 6-minute run, star run, standing long jump, and ball-push test. The pandemic effect interacted with sex, indicating that only boys exhibited a negative pandemic effect on 6-minute run performance, and only girls exhibited a negative pandemic effect on ball-push test performance. The pandemic’s main effect on static balance was insignificant, but partial effects suggested that girls showed a small improvement at the critical date.

The effects of the pandemic on PF have been investigated in previous studies and differed between regions, samples, and specific fitness components^24,25,27,29–38^. Our findings aligned with previous studies reporting negative pandemic effects on cardiorespiratory endurance^24,29,30,31,36,37,38^, PowerLOW^24,28,34,35^, and powerUP^28,34,38^in school children. For instance, in the Federal State of Brandenburg, Germany, third-graders completed the same six fitness tests as children in the present study. They exhibited small negative pandemic effects in the 6-minute run, star run, 20-meter sprint, and ball-push test. However, the standing long jump performance did not significantly decrease at the pandemic onset^38^, and some other studies even reported gains in cardiorespiratory endurance^35^, PowerLOW^28,34,36^, and powerUP^29^ after the onset of the pandemic.

We did not have explicit hypotheses regarding the interactions between sex and the pandemic for the 6-minute run, ball-push test, and one-legged stance test. However, based on the AIC, we incorporated these interactions into the LMM to investigate any significant effects. Conversely, the more conservative BIC showed that including these interactions did not enhance the model fit, which might suggest overfitting.

In the present study, the 6-minute run and 20-meter sprint performance were lower during the pandemic than in pre-pandemic years. However, this decrease was confounded by negative linear secular trends in both pre-pandemic and pandemic cohorts (see Fig. 4). Indeed, the negative pre-pandemic slope for speed was unexpected, given that, for example, speed had increased for Brandenburg children over the last decades before the COVID-19 pandemic^18,54^. Additionally, in contrast to the continued decrease in the 6-minute run observed in the present study, data from Brandenburg also indicated a plateau in 6-minute run performance in the years before the pandemic, which was followed by a step-down at the critical date and stagnation at low levels in recent years^36^.

The correlation parameters related to the random factor school indicated that “fitter” schools exhibited larger negative impacts from the pandemic at the critical date. Additionally, these “fitter” schools were less likely to show declines in performance before the pandemic. In contrast, schools with a positive cohort trend before the pandemic were more likely to be affected by a negative pandemic impact. The significant fitness losses related to the pandemic can be referred to as a “reversed Matthew effect,” where schools with higher average fitness had greater losses. These differences between schools indicate that varying circumstances and school focuses affect PF. Thus, the closure of schools worsened the fitness development of children who attended schools with favorable PF circumstances.

In contrast, a masked effect from closed schools, which provide poor PF-promoting circumstances, lowered the pandemic’s overall impact in the present study’s available data. However, our results align with findings from the German federal states of Brandenburg and Berlin, indicating more pronounced fitness losses in “fitter” schools and schools with a higher socioeconomic background^35,36^. Since parental socioeconomic status and access to sports opportunities impact activity levels and physical fitness^35,95,96^, larger pandemic-related fitness losses in well-to-do areas highlight the effect of PF-promoting circumstances on children’s fitness development. In contrast, it strongly argues for improving infrastructural sports opportunities and combating child poverty. This is especially important as cardiorespiratory endurance predicts children’s physical^10,11^and cognitive well-being^97,98^.

## Limitations

The present study was based on quasi-experimental cross-sectional data across seven cohorts of children in third grade of primary school, covering the COVID-19 pandemic as a “natural experiment,” meaning that caution is warranted when interpreting results, especially when interested in cause-effect relationships^99,100^. In our study, the effects of the COVID-19 pandemic were confounded with secular fitness trends^18,36^. However, we adjusted for these confounds by using a regression discontinuity design^70,71^, which provided a conservative estimate of the pandemic effects on children’s performance in six fitness tests.

Several aspects limit the generalizability of the results. Tests included in the battery had to be appropriate for a school environment, allowing physical education teachers to perform them using only equipment typically found in a school gym. The fitness tests were carried out in groups within a school gym, leading to a less controlled setting that may be influenced by group dynamics and various school-specific factors, potentially impacting children’s performance compared to individual evaluations in a laboratory. Another related limitation is the presumption that independent research teams provide more accurate and valid assessments than physical education teachers, whose evaluations may be influenced by their familiarity with the children’s abilities. Nevertheless, there are counterarguments; for instance, physical education teachers might possess greater expertise in administering these tests to groups of primary school children than assessment teams made up of university students or research assistants.

A latent definition of each physical fitness component should be made with at least two test items^101^. Still, due to time constraints associated with school assessments, our test battery assesses each fitness component using only one fitness test. Further, both weight-bearing (6-minute run, star run, 20-meter sprint, and standing long jump) and non-weight-bearing tests (ball-push test and one-legged-stance) were included in the test battery, which becomes visible in their distinctly different zBMI functions. Due to the better performance of overweight or obese children in non-weight-bearing muscular fitness tests^40,94,102^, some studies have used relative strength (strength-to-body-mass ratios) to predict health outcomes^103^. It is likely that relative powerUP, adjusted for children’s body mass, would exhibit a similar relationship with outcomes such as percent body fat or waist circumference as weight-bearing tests of muscle power. The results of the present study are thus limited to the six specific tests used and cannot be generalized to different tests of the same six fitness components.

In the present study, children’s mass and height were not measured at school but were provided voluntarily by 92.3% of the parents. Such self-reported information may be biased, but there is evidence that such information is still valid and reliable^51–53^. In the present data, children with information about body constitution scored significantly higher on four tests (6-minute run, star run, 20-meter sprint, and standing long jump). However, there were no significant interactions with test, age, or sex (see OSF repository https://osf.io/ztyfp/for a synopsis^104^). Moreover, we reanalyzed data from Golle et al.^85^, who used measures, not self-reports, of 240 children’s body constitution indices to predict performances in tests covering the same fitness components (except for a test assessing flexibility instead of static balance). This reanalysis revealed the same differences in curvilinear profiles between the PF components with BMI, mass, and height as reported here (see OSF repository https://osf.io/ztyfp/for a synopsis^105^).

## Conclusion and outlook

The present study met three objectives. First, it closely replicated third-graders’ age and sex differences in PF components (see Fig. 2). Replication studies with very large samples are paramount for the credibility of behavioral and social sciences. The present study, along with the reference project, provides a solid foundation for future research monitoring secular trends of children’s physical fitness and addressing potential fitness declines.

Second, with additional information about children’s body constitution, we documented the specificity of curvilinear relations between body constitution and different components of physical fitness as well as their dependency on sex and age within the ninth year of life (see Figs. 3 and 4). The preciseness of these results provides benchmark targets for accounts with alternative physiological and biomechanical indicators.

Third, the span of cohorts from 2017 to 2023 permitted the assessment and replication of the COVID-19 pandemic’s effects on PF components against the background of secular trends. We conclude that our results largely, but not completely, agree with other research reporting small but reliable negative associations with performance in running tasks (see Fig. 5).

These results have implications for educational and social settings. First, we conclude that school-based fitness assessments are well-suited for developing fitness norms. Qualified physical education teachers can administer fitness tests and yield large representative samples (or even population statistics). They may also motivate consideration of body constitution in norm-referenced tests that currently are based only on sex and age categories.

Second, results of school-based assessments may reveal temporal and regional fitness trends that can be used as proxies for a population’s health status. Small negative fitness changes can serve as early warning signals that trigger evidence-based countermeasures by policymakers, schools, and other experts^106^.

Third, school-based fitness assessments may guide prevention programs and raise awareness of the importance of fitness for healthy development across the lifespan^3,10,107^. For example, the results on pronounced COVID-19 pandemic-related performance declines in “fitter” schools underscore the importance of access to physical activity resources and reveal school disparities that need to be addressed. The results should motivate an extension of sports infrastructure and access to physical activity opportunities for all children to promote healthier lifestyles and enhance their future quality of life.

## Electronic supplementary material

Below is the link to the electronic supplementary material.

Supplementary Material 1

## Data Availability

Data as well as R and Julia scripts are available in the Open Science Framework (OSF) repository: https://osf.io/ztyfp/.
