# Supplementary material for: Impact of age, sex, body constitution, and the COVID-19 pandemic on the physical fitness of 38,084 German primary school children
Source: Sci Rep. 2025 Apr 2;15:11300. doi: 10.1038/s41598-025-95461-5 (PMC11965452; doi:10.1038/s41598-025-95461-5)
Supplement: Supplementary file 1 — Supplementary Material 1 [file 41598_2025_95461_MOESM1_ESM.pdf]

Supplementary Material

Impact of Age, Sex, Body Constitution, and the COVID-19 Pandemic on the Physical Fitness of 38,084 German Primary School Children.

Florian Bähr[1], Toni Wöhrl[1], Paula Teich[2], Christian Puta[345] & Reinhold Kliegl[2]

[1]University of Erfurt, Faculty of Educational Sciences, Division of Sports and Movement Sciences, Erfurt, Germany

[2]University of Potsdam, Faculty of Human Sciences, Department of Sports and Health Sciences, Potsdam, Germany

[3]Friedrich-Schiller-University of Jena, Department of Sports Medicine and Health Promotion, Jena, Germany

[4]Center for Interdisciplinary Prevention of Diseases related to Professional Activities, Jena, Germany

[5]Center for Sepsis Control and Care (CSCC), Jena University Hospital/ Friedrich-Schiller-University Jena, Jena, Germany

Corresponding author

Florian Bähr

Universität Erfurt

Campus

Nordhäuser Straße 63

99089 Erfurt

florian.baehr@uni-erfurt.de

Note: Data, along with Julia and R scripts for reproducing figures and analyses, are available at the OSF repository (https://osf.io/ztyfp/).

Supplementary Table 1. LMM fixed-effect estimates and test statistics

Test	Coef	Est	SE	z	p
Endurance	sex	0.4823	0.0161	29.9814	0.0000
	age	0.0925	0.0143	6.4889	0.0000
	zBMI	-0.6058	0.0354	-17.1050	0.0000
	zBMI2	-0.1901	0.0046	-41.3501	0.0000
	zBMI3	-0.0395	0.0017	-22.7818	0.0000
	sex x zBMI	-0.0720	0.0082	-8.7815	0.0000
	sex x zBMI2	-0.0457	0.0053	-8.5590	0.0000
	age x zBMI	-0.0869	0.0123	-7.0402	0.0000
	zHeight	-0.2269	0.0242	-9.3575	0.0000
	zHeight2	0.0008	0.0044	0.1910	0.8485
	zMass	0.4312	0.0484	8.9168	0.0000
	cvd	-0.0640	0.0291	-2.1950	0.0282
	pre CD	-0.0695	0.0106	-6.5372	0.0000
	post CD	-0.0079	0.0103	-0.7663	0.4435
	cvd x sex	-0.0475	0.0190	-2.5022	0.0123
	cvd x zBMI	0.0072	0.0085	0.8484	0.3962
Coordination	sex	0.2251	0.0179	12.5752	0.0000
	age	0.3583	0.0158	22.7114	0.0000
	zBMI	-0.2634	0.0397	-6.6354	0.0000
	zBMI2	-0.1106	0.0051	-21.7393	0.0000
	zBMI3	-0.0245	0.0019	-12.8107	0.0000
	sex x zBMI	-0.0414	0.0091	-4.5544	0.0000
	sex x zBMI2	-0.0299	0.0059	-5.0834	0.0000
	age x zBMI	-0.0771	0.0136	-5.6547	0.0000
	zHeight	-0.0314	0.0272	-1.1544	0.2484
	zHeight2	-0.0116	0.0049	-2.3456	0.0190
	zMass	0.1760	0.0543	3.2428	0.0012
	cvd	-0.1804	0.0295	-6.1130	0.0000
	pre CD	-0.0060	0.0118	-0.5090	0.6108
	post CD	0.0263	0.0107	2.4597	0.0139
	cvd x sex	0.0320	0.0211	1.5205	0.1284
	cvd x zBMI	0.0010	0.0094	0.1023	0.9185

Test	Coef	Est	SE	z	p
Speed	sex	0.2995	0.0171	17.5386	0.0000
	age	0.2364	0.0151	15.6559	0.0000
	zBMI	-0.5535	0.0375	-14.7701	0.0000
	zBMI2	-0.1852	0.0049	-38.1061	0.0000
	zBMI3	-0.0418	0.0018	-22.6841	0.0000
	sex x zBMI	-0.0519	0.0087	-5.9867	0.0000
	sex x zBMI2	-0.0483	0.0057	-8.5375	0.0000
	age x zBMI	-0.0813	0.0130	-6.2307	0.0000
	zHeight	-0.1622	0.0256	-6.3284	0.0000
	zHeight2	-0.0050	0.0046	-1.0859	0.2775
	zMass	0.4273	0.0512	8.3493	0.0000
	cvd	-0.0303	0.0292	-1.0374	0.2995
	pre CD	-0.0358	0.0108	-3.3061	0.0009
	post CD	-0.0270	0.0105	-2.5667	0.0103
	cvd x sex	0.0147	0.0201	0.7288	0.4661
	cvd x zBMI	0.0187	0.0090	2.0807	0.0375
PowerLOW	sex	0.3428	0.0172	19.8884	0.0000
	age	0.2681	0.0152	17.5939	0.0000
	zBMI	-0.6171	0.0384	-16.0893	0.0000
	zBMI2	-0.1918	0.0049	-39.0197	0.0000
	zBMI3	-0.0407	0.0019	-21.8454	0.0000
	sex x zBMI	-0.0435	0.0088	-4.9612	0.0000
	sex x zBMI2	-0.0312	0.0057	-5.4429	0.0000
	age x zBMI	-0.0989	0.0132	-7.4946	0.0000
	zHeight	-0.1688	0.0263	-6.4244	0.0000
	zHeight2	0.0054	0.0048	1.1352	0.2563
	zMass	0.4986	0.0524	9.5133	0.0000
	cvd	-0.0748	0.0281	-2.6623	0.0078
	pre CD	0.0208	0.0097	2.1477	0.0317
	post CD	-0.0021	0.0105	-0.1988	0.8424
	cvd x sex	0.0258	0.0203	1.2711	0.2037
	cvd x zBMI	-0.0067	0.0091	-0.7374	0.4609

Test	Coef	Est	SE	z	p
PowerUP	sex	0.6117	0.0158	38.6680	0.0000
	age	0.5731	0.0140	40.8815	0.0000
	zBMI	-0.0495	0.0348	-1.4221	0.1550
	zBMI2	-0.0433	0.0045	-9.5947	0.0000
	zBMI3	-0.0129	0.0017	-7.5548	0.0000
	sex x zBMI	0.0076	0.0080	0.9398	0.3473
	sex x zBMI2	-0.0096	0.0052	-1.8279	0.0676
	age x zBMI	-0.0126	0.0121	-1.0400	0.2983
	zHeight	0.1913	0.0238	8.0472	0.0000
	zHeight2	0.0054	0.0043	1.2461	0.2127
	zMass	0.2087	0.0475	4.3964	0.0000
	cvd	-0.1652	0.0270	-6.1290	0.0000
	pre CD	0.0443	0.0096	4.5944	0.0000
	post CD	0.0515	0.0102	5.0377	0.0000
	cvd x sex	0.0589	0.0186	3.1607	0.0016
	cvd x zBMI	-0.0022	0.0083	-0.2659	0.7903
Balance	sex	-0.2097	0.0182	-11.5085	0.0000
	age	0.1405	0.0161	8.7498	0.0000
	zBMI	-0.1973	0.0399	-4.9428	0.0000
	zBMI2	-0.0775	0.0052	-14.9240	0.0000
	zBMI3	-0.0191	0.0020	-9.7671	0.0000
	sex x zBMI	-0.0004	0.0093	-0.0479	0.9618
	sex x zBMI2	-0.0074	0.0060	-1.2280	0.2194
	age x zBMI	-0.0602	0.0139	-4.3312	0.0000
	zHeight	-0.1095	0.0273	-4.0087	0.0001
	zHeight2	0.0004	0.0049	0.0758	0.9396
	zMass	0.1737	0.0545	3.1857	0.0014
	cvd	0.0571	0.0296	1.9308	0.0535
	pre CD	-0.0062	0.0110	-0.5618	0.5742
	post CD	-0.0111	0.0108	-1.0285	0.3037
	cvd x sex	-0.0498	0.0214	-2.3250	0.0201
	cvd x zBMI	-0.0362	0.0096	-3.7685	0.0002

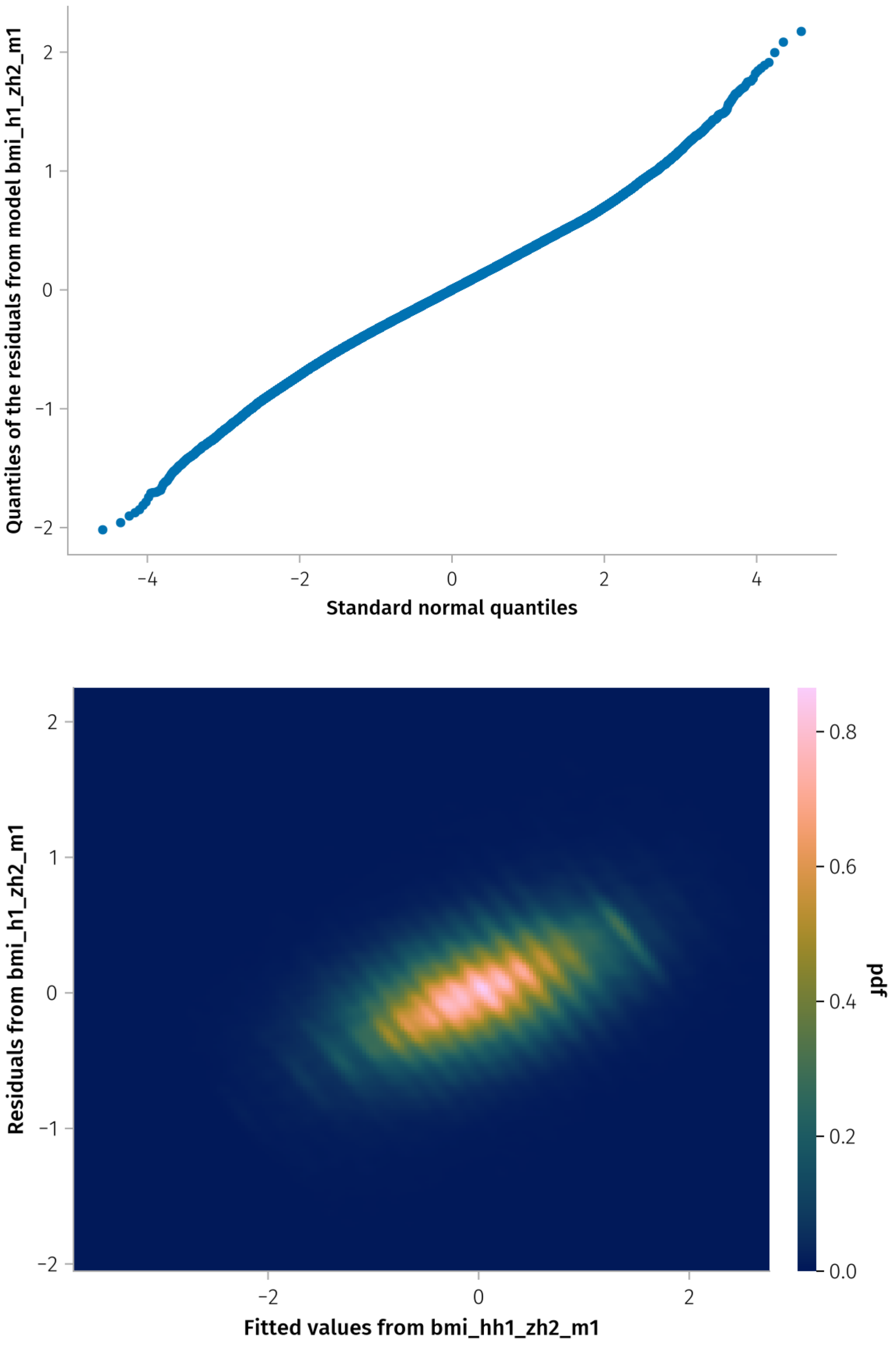

Supplementary Figure 1. (A) Quantile-quantile plot of the residuals for model m1 versus a standard normal. (B) Heatmap of residuals versus fitted values for model

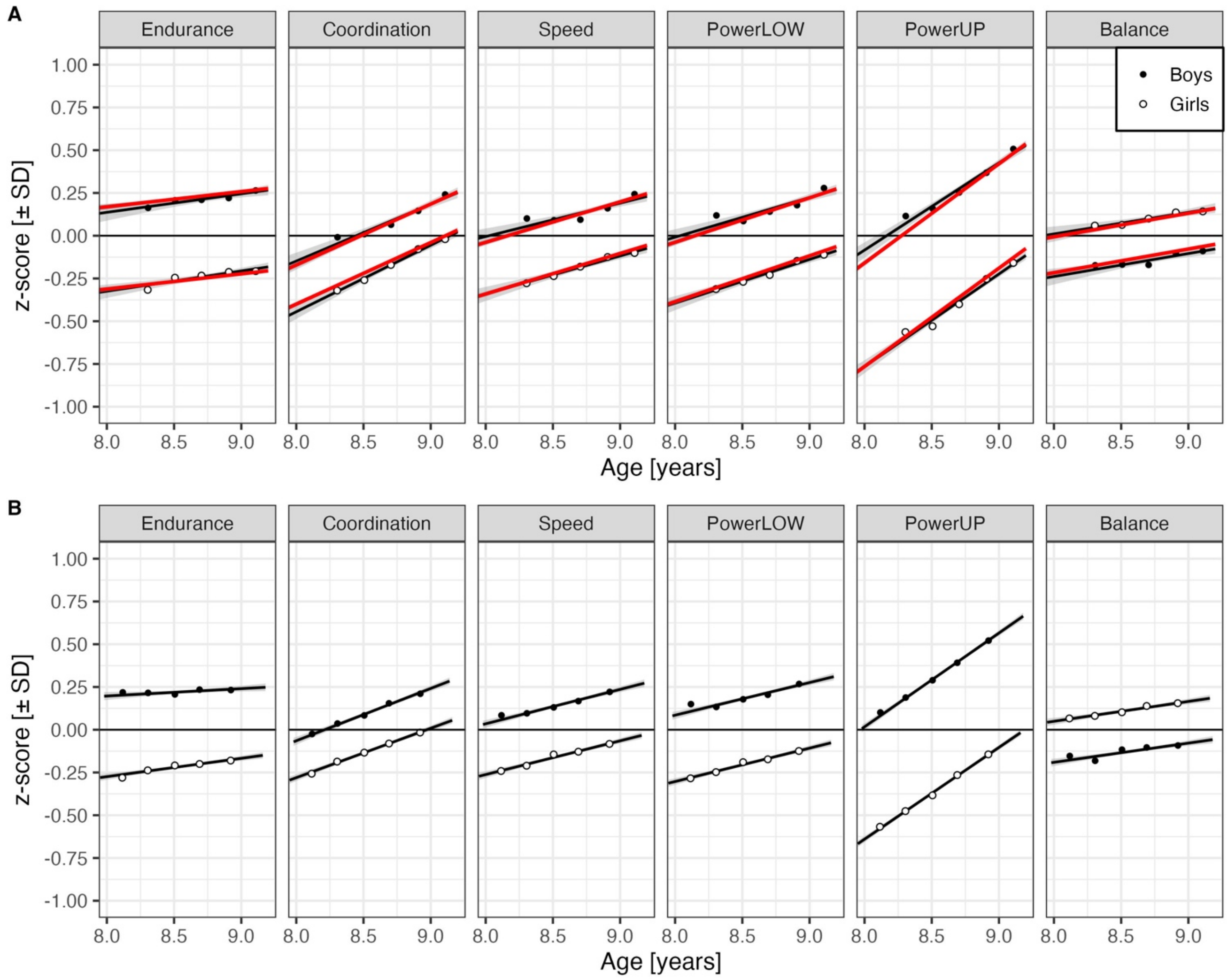

Supplementary Figure 2. Age and sex effects on six physical fitness components for third-grade elementary school children from (A) 2017-2023 cohorts in Thuringia (N=38,084 children) and (B) 2016-2022 cohorts in Brandenburg (N=98,510 children). Endurance = 6-minute run, Coordination = star run, Speed = 20-meter sprint, PowerLOW = standing long jump, PowerUP = ball-push test, and Balance = one-legged-stance. Points represent means for 0.20-year-wide bins; black smooths indicate linear fits to observations; red smooths represent fits to partial effects. Error bands for smooths show 95% confidence intervals.

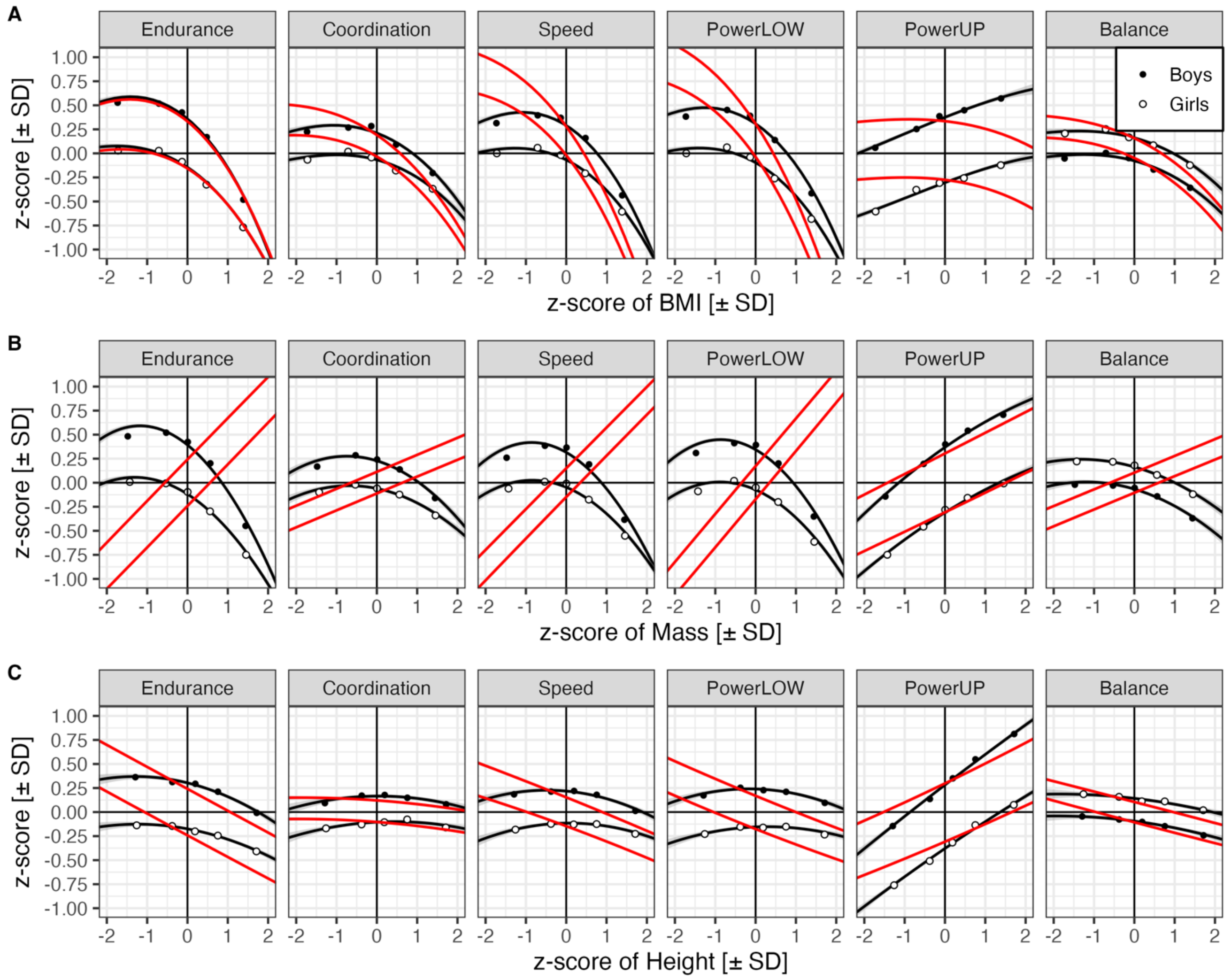

Supplementary Figure 3. Zero-order fixed effects illustrate the interaction of sex with (A) cubic-trend effects of zBMI, (B) quadratic zMass, and (C) quadratic zHeight fitted to observations; the shaded areas represent 95% confidence intervals (CIs). Endurance = 6-minute run, Coordination = star run, Speed = 20-meter sprint, PowerLOW = standing long jump, PowerUP = ball-push test, and Balance = one-legged-stance. The points indicate observed z-score means for bins containing approximately equal-sized numbers of children. The interaction with sex shows significance in both the linear and quadratic trends of zBMI.

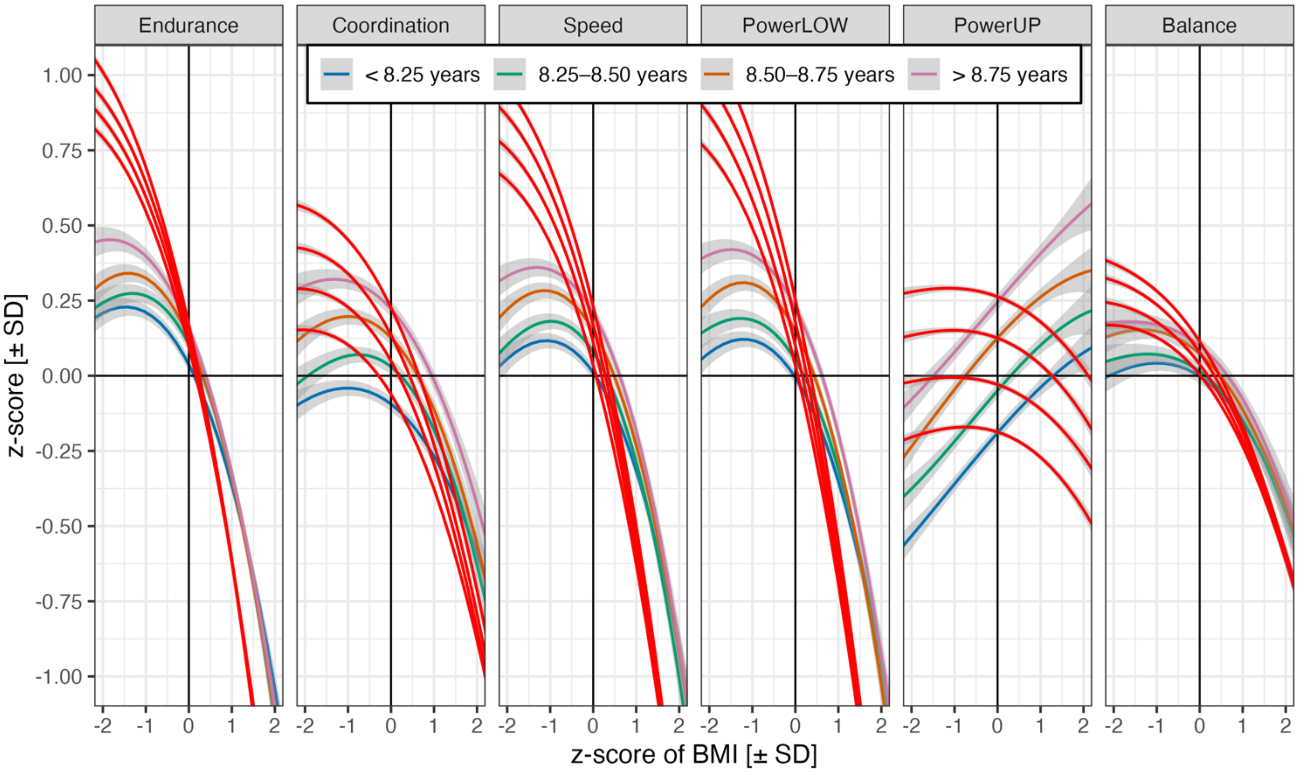

Supplementary Figure 4. Zero-order fixed effects for the interaction between age and cubic-trend effects of zBMI are fitted to observations (color scale) and partial effects (red) for four age groups; shaded areas represent 95% confidence intervals (CIs). Children were grouped into four age categories for visualizing the interaction; in the linear mixed model (LMM), age was treated as a continuous linear covariate and significantly interacted with the linear trend of zBMI. Endurance = 6-minute run, Coordination = star run, Speed = 20-meter sprint, PowerLOW = standing long jump, PowerUP = ball-push test, and Balance = one-legged-stance.

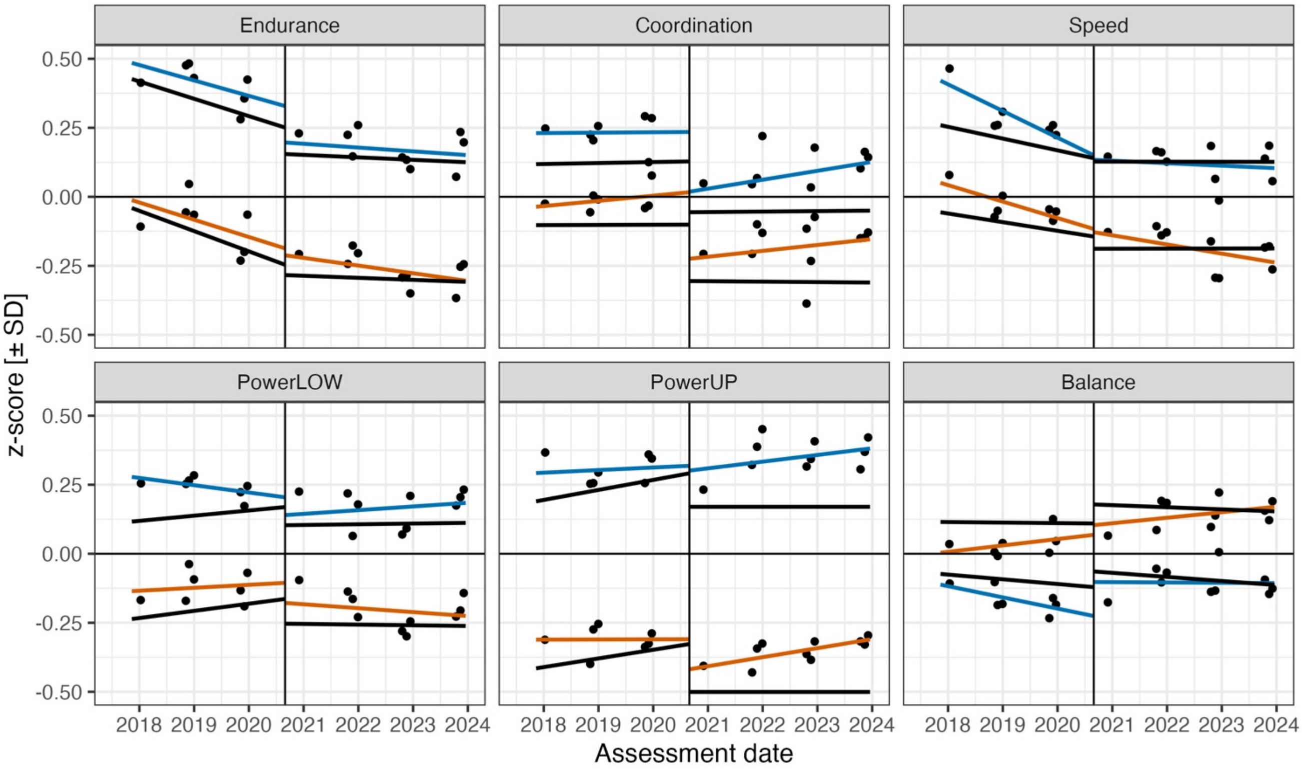

Supplementary Figure 5. Zero-order relations for the regression of physical fitness on the assessment date (2017-11-20 to 2023-12-21) and the critical date for testing the COVID-19 effect on August 30, 2020 (i.e., the vertical line marks the first day of school in third grade for the 2020 cohort of boys, blue line, and girls, red line). The black dots represent the observed z-score means for bins of assessment dates with at least 1,000 children. The black lines indicate the partial effects of the COVID-19 and sex interaction. Endurance = 6-minute run, Coordination = star run, Speed = 20-meter sprint, PowerLOW = standing long jump, PowerUP = ball-push test, and Balance = one-legged-stance.
